# Universal Live-Attenuated Influenza Vaccine Candidates Expressing Multiple M2e Epitopes Protect Ferrets against a High-Dose Heterologous Virus Challenge

**DOI:** 10.3390/v13071280

**Published:** 2021-06-30

**Authors:** Daria Mezhenskaya, Irina Isakova-Sivak, Victoria Matyushenko, Svetlana Donina, Andrey Rekstin, Konstantin Sivak, Kirill Yakovlev, Anastasia Katelnikova, Kirill Kryshen, Valery Makarov, Larisa Rudenko

**Affiliations:** 1 Department of Virology, Institute of Experimental Medicine, 197376 Saint Petersburg, Russia; dasmez@iemspb.ru (D.M.); matyshenko@iemspb.ru (V.M.); sveta.donina@gmail.com (S.D.); arekstin@yandex.ru (A.R.); vaccine@mail.ru (L.R.); 2 Department of Preclinical Trials, Smorodintsev Research Institute of Influenza, 197376 Saint Petersburg, Russia; konstantin.sivak@influenza.spb.ru (K.S.); kirikus-fly@yandex.ru (K.Y.); 3 Department of Toxicology and Microbiology, Institute of Preclinical Research Ltd., 188663 Saint Petersburg, Russia; katelnikova.ae@doclinika.ru (A.K.); kryshen.kl@doclinika.ru (K.K.); makarov.vg@doclinika.ru (V.M.)

**Keywords:** universal influenza vaccine, live attenuated influenza vaccine, M2e epitope, ferret model, recombinant vaccine, cross-protection

## Abstract

The development of an influenza vaccine with broad protection and durability remains an attractive idea due to the high mutation rate of the influenza virus. An extracellular domain of Matrix 2 protein (M2e) is among the most attractive target for the universal influenza vaccine owing to its high conservancy rate. Here, we generated two recombinant live attenuated influenza vaccine (LAIV) candidates encoding four M2e epitopes representing consensus sequences of human, avian and swine influenza viruses, and studied them in a preclinical ferret model. Both LAIV+4M2e viruses induced higher levels of M2e-specific antibodies compared to the control LAIV strain, with the LAIV/HA+4M2e candidate being significantly more immunogenic than the LAIV/NS+4M2e counterpart. A high-dose heterosubtypic influenza virus challenge revealed the highest degree of protection after immunization with LAIV/HA+4M2e strain, followed by the NS-modified LAIV and the classical LAIV virus. Furthermore, only the immune sera from the LAIV/HA+4M2e-immunized ferrets protected mice from a panel of lethal influenza viruses encoding M genes of various origins. These data suggest that the improved cross-protection of the LAIV/HA+4M2e universal influenza vaccine candidate was mediated by the M2e-targeted antibodies. Taking into account the safety profile and improved cross-protective potential, the LAIV/HA+4M2e vaccine warrants its further evaluation in a phase I clinical trial.

## 1. Introduction

Influenza viruses cause acute respiratory tract infections that occur periodically in the form of epidemics and pandemics and remain a major unresolved public health problem worldwide. Annual influenza epidemics cause 3 to 5 million cases of severe respiratory diseases, up to 650,000 of which are fatal [1]. Despite the decreased influenza activity in the 2020–2021 season due to the measures taken to combat the new coronavirus disease COVID-19 [2], influenza viruses continue circulating in human and animal reservoirs with the potential to cause severe epidemics. Vaccination is by far the most effective measure to prevent the spread of influenza. However, seasonal vaccines induce primarily strain-specific immunity and are not effective against drifted seasonal or a new pandemic influenza virus. One of the possible ways to prevent epidemics caused by antigenically evolved viruses or pandemics caused by new influenza viruses could be the creation of a universal influenza vaccine, i.e., an influenza vaccine that has a broader protective effect than the seasonal vaccine [3,4]. One of the viral antigens which is most often used to design a universal influenza vaccine is an extracellular domain of matrix 2 protein (M2e) due to its high conservancy rate among all influenza A viruses. However, the M2e fragment itself is a weak immunogen due to its size, the small number of copies per virion, and also due to the shielding effect of the larger surface proteins of the influenza A virus. Therefore, multiple strategies have been proposed to increase the immunogenic and protective potential of this small protein [5]. Previously, we have developed M2e-based universal influenza vaccine prototypes by genetic fusion of four M2e tandem repeats to the N-terminus of hemagglutinin (HA) subunit 1 and rescue of live attenuated influenza vaccine (LAIV) viruses based on a cold-adapted A/Leningrad/134/17/57 backbone. Mouse studies confirmed that these modified LAIVs were capable of inducing higher levels of M2e-specific antibodies compared to classical LAIVs, and these antibodies were associated with enhanced protection against a panel of heterologous and heterosubtypic influenza viruses [6,7]. To further optimize these vaccine candidates, we adjusted the consensus amino acid sequences of the M2e epitopes according to our comprehensive analysis of all influenza A viruses circulating in human and animal reservoirs [5]. The new 4M2e construct consisted of four 23 amino acids-long M2e epitopes separated by flexible linkers, including consensus human (classical human, hM2e), swine (M2 of classical swine lineage, sM2e), avian/swine (M2 of swine influenza viruses derived from Eurasian avian H1N1, a/sM2e), and human/swine (M2 of pandemic H1N1, h/sM2e) M2 lineages. This construct was inserted in full-length HA or truncated NS1 genes of an H3N2 LAIV virus and two recombinant LAIV+4M2e viruses were assessed in a pre-clinical ferret model. Ferrets are considered the best test system for studying influenza in humans, as they are most susceptible to infection with virulent strains of influenza virus when administered intranasally and their infection proceeds as in humans with similar clinical symptoms [8].

## 2. Materials and Methods

### 2.1. Cells, Viruses and Proteins

African green monkey kidney (Vero) cells were cultured in OptiPRO serum-free media supplemented with antibiotic-antimycotic and GlutaMAX (all from Thermo Fisher Scientific, Waltham, MA, USA) at 37 °C in the atmosphere of 5% CO_2_. Madin–Darby Canine Kidney (MDCK) cells were maintained in DMEM media with 10% fetal bovine serum (FBS) and 1x antibiotic-antimycotic (all from Thermo Fisher Scientific) at the same conditions as Vero cells.

An H3N2 LAIV reassortant virus carrying HA and NA genes of a recent H3N2 virus and six remaining genes of A/Leningrad/134/17/57 (H2N2) cold-adapted master donor virus was used as a viral vector for designing more broadly protective M2e-based vaccines. A set of dual-promoter plasmids encoding all genes of A/Leningrad/134/17/57 (H2N2) and A/Puerto Rico/8/34 (H1N1) viruses were described earlier [9]. To rescue universal vaccine prototypes, as well as a control LAIV strain, wild-type HA and NA gene of A/Switzerland/9715293/2013 (H3N2) virus were cloned into pCIPolISapIT vector. To rescue a panel of recombinant PR8-based viruses, M genes of A/duck/Potsdam/1402-6/1986 (H5N2) and A/Aichi/2/68 (H3N2) viruses were also cloned into pCIPolISapIT vector by standard procedures. To rescue a PR8-based virus with M gene of swine-origin lineage, site-directed mutagenesis of A/Leningrad/134/17/57 was used to generate the consensus M2e sequence. This gene was also cloned into pCIPolISapIT vector to rescue the corresponding recombinant virus.

**Figure 1:**
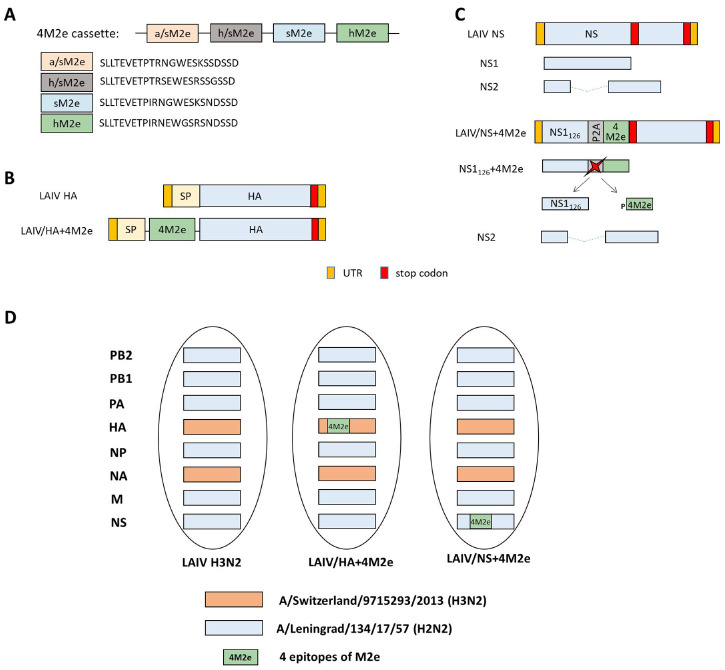
Overview of LAIV viruses used in this study. (**A**) Scheme of the 4M2e cassette. Flexible Gly-linkers were used to join the M2e epitopes. (**B**) Scheme of the chimeric HA gene containing the 4M2e cassette. (**C**) Scheme of the chimeric truncated NS1 gene containing the 4M2e cassette. (**D**) Genome composition of the LAIV viruses used in this study. hM2e: human M2e lineage; sM2e: swine M2e lineage; a/sM2e: avian/swine M2e lineage; h/sM2e: human/swine M2e lineage, as described in [5]. 2A: porcine teschovirus-1 P2A self-cleavage site (GSGATNFSLLKQAGDVEENPG↓P).

A DNA fragment encoding 4 M2e epitopes separated by flexible linkers (Figure 1A) was chemically synthesized by Evrogen Ltd. (Moscow, Russia). The 4M2e construct was cloned from the N-terminus of the HA1 subunit and also in-frame of the NS1 gene truncated to 126 amino acids, via the P2A self-cleavage site, as previously described [7,10] (Figure 1B,C). Briefly, the BsmBI restriction sites were inserted between the signal peptide and the HA1 subunit of the H3 HA molecule and at the residue NS1_126_ of the A/Leningrad/134/17/57 NS gene using standard gene engineering approaches. Then, the 4M2e cassette was am amplified using sequence-specific primers with extended ends containing sites for the BsmBI restriction enzyme. Following restriction of the modified plasmids and the amplified insert with subsequent ligation resulted in generation of the HA+4M2e and NS+4M2e recombinant genes.

Two recombinant H3N2 LAIV viruses, LAIV/HA+4M2e and LAIV/NS+4M2e were rescued by electroporation of Vero cells using Neon transfection system (Thermo Fisher Scientific, USA), amplified in eggs and stored in single-use aliquots at −70 °C.

Influenza A virus A/South Africa/3626/2013 (pH1N1) (S.A. H1N1) was from the influenza virus repository of the Department of Virology, Institute of Experimental Medicine (Saint Petersburg, Russia). A mouse-adapted A/California/7/2009 (pH1N1) virus was obtained from the repository of respiratory viruses of Smorodintsev Research Institute of Influenza (Saint Petersburg, Russia).

A recombinant 3M2e protein expressed in *E. coli* was kindly provided by Dr. Andris Kazaks (Latvian Biomedical Research and Study Centre, Riga, Latvia). The sequence of this protein was reported earlier [11].

### 2.2. In Vitro Studies

#### 2.2.1. Expression of M2e Epitopes by Recombinant LAIV+4M2e Viruses

To assess the expression of M2e epitopes within an infected cell, MDCK cell monolayers were infected with studied viruses at various multiplicities of infection (MOI, number of TCID_50_ per cell): 1.0, 0.2, 0.04, and 0.008. The next day, the cells were fixed with 80% acetone, washed twice with PBS supplemented with 3% tween (PBST) and blocked with 5% non-fat dry milk in PBS. Then the cells were incubated with primary 14C2 antibody, followed by addition of secondary anti-mouse IgG antibody (Abcam, Cambridge, UK). The antibody binding was detected with 1-Step Ultra TMB-ELISA Substrate Solution (Thermo Fisher Scientific) and the color development was stopped by the addition of 1M H_2_SO_4_. The absorbance was measured at 450 nm using an xMark Microplate Spectrophotometer (BioRad, Hercules, CA, USA).

#### 2.2.2. Growth Characteristics of LAIV Viruses

Growth characteristics of recombinant LAIV viruses were assessed in eggs and MDCK cells. Infectious titers in eggs were determined by end-point titration and titer calculation by the Reed and Muench method [12] and expressed in log_10_ 50% egg infectious doses (log_10_EID_50_/mL). The virus was considered temperature-sensitive (ts) if the titer at 38 °C was reduced ≥5.0 log_10_EID_50_ compared to 33 °C. The virus was considered cold-adapted if the titer at 26 °C is reduced by ≤3.0 log_10_EID_50_ compared to 33 °C.

### 2.3. Animals, Immunization and Challenge

Male ferrets (aged 5–6 months and weighing 1.1–1.9 kg at the beginning of the experiment) were supplied by Scientific-Production Organization House of Pharmacy JSC (St. Petersburg, Russia). All animal experiments were approved by the Local Ethics Committee of the Institute of Preclinical Research Ltd., Saint Petersburg (Approval No. 4.51/20 from 11 September 2020).

All inoculations, nasal washes and blood sample collections were performed with the animal under short-term anesthesia induced by intramuscular injection of Zoletil 100, 12.5 mg/kg of body weight; every effort was made to minimize suffering. At the end of the study, animals were euthanized with an overdose of Zoletil-xylazine combination.

Before the study all ferrets were prescreened by hemagglutination inhibition test (HAI) to verify their negativity to circulating H1N1 and H3N2 human influenza viruses.

For the serum transfer experiments six to eight-week-old female C57BL/6J mice were purchased from Stolbovaya animal breeding nursery laboratory (Moscow region, Russia). Mice were anesthetized with isoflurane and all efforts were made to minimize animal suffering.

### 2.4. Replication of LAIV Viruses in the Upper Respiratory Tract

To determine the ability of the classical and recombinant LAIV viruses to replicate in the upper respiratory tract (URT) of immunized ferrets, nasal wash samples were collected from each animal on days 1, 2, 3 and 4 after each vaccine dose. 1 mL of sterile PBS was administered to one nostril of anesthetized ferret and the fluid excreting from the other nostril was collected using a petri dish. Ten-fold dilutions of the nasal wash (NW) fluids were inoculated into eggs and were incubated for 2 days at 33 °C and positive eggs were determined by HA assay using chicken red blood cells. Viral titers were calculated using Reed and Muench method and expressed in log_10_EID_50_/mL.

### 2.5. Safety Assessment

Assessment of the safety of LAIV candidates included several parameters, such as analysis of body weight and temperature of animals, data of clinical observations, as well as pathological examination of the respiratory tract of animals after immunization. Analysis of temperature, body weight of ferrets and clinical symptoms was carried out within 28 days of experiment, i.e., until day 7 after the second dose. At this time point, three animals per group were euthanized and subjected to autopsy. During autopsy, the external state of the body, chest and abdominal cavity with organs and tissues were examined in detail. Organ tissues (heart, trachea, lungs, liver, kidneys, adrenal glands, spleen, thymus, regional lymph node) were subjected to histological examination. The local irritant effect was assessed by histological analysis of the site of vaccine administration—the mucous membrane of the nasal cavity after decalcifying treatment. Tissues were fixed in a 10% buffered formalin solution and subjected to standard processing on an automatic Histo-Tek VP1 (Sakura, Alphen aan den Rijn, The Netherlands) histoprocessor with subsequent conclusion on Histomix medium (BioVitrum, St. Petersburg, Russia). Sections were made from paraffin blocks and were stained with hematoxylin and eosin, as well as with alcian blue dyeing for mucopolysaccharides (for the nasal cavity). Histological preparations were examined in a light microscope DM1000 (Leica, Wetzlar, Germany).

### 2.6. Assessment of Immune Responses

#### 2.6.1. Hemagglutination Inhibition (HAI) Assay

HAI antibody titers in ferret sera were determined by the standard protocol described elsewhere [13]. Briefly, ferrets’ sera were treated with receptor destroying enzyme (RDE, Sigma Aldrich, St. Louis, MO, USA) at 1:3 ratio and incubated for 16 h at 37 °C. Then, the treated samples were inactivated for 30 min at 56 °C. Serial 2-fold dilutions of each serum sample were incubated with four HA units of S.A. H1N1 and H3N2 LAIV viruses for 30 min and then mixed with 0.5% chicken red blood cells in the v-bottom 96-well microtiter plates. HAI titers were determined as the last serum dilution with complete inhibition of hemagglutination.

#### 2.6.2. ELISA

Serum IgG antibody titers in ferret sera were determined on day 21 after each vaccine dose using enzyme-linked immunosorbent assay (ELISA). High-sorbent 96-well plates (Greiner Bio-One, Frickenhausen, Germany) were coated with 50 ng/well of either S.A. H1N1 or H3N2 LAIV sucrose-purified whole virus or recombinant 3M2e protein in a carbonate-bicarbonate buffer, in a volume of 50 µL per well at 4 °C overnight.

Two-fold dilutions of sera were prepared starting from 1:20 (for M2e antigen) or 1:200 (for H3N2 LAIV and S.A. H1N1 antigens) and added to the coated wells and incubated for 1 h at 37 °C, followed by incubation with anti-ferret IgG conjugated to horseradish peroxidase (Sigma). Antibody binding was detected with 1-Step Ultra TMB-ELISA Substrate Solution after incubation for 15 min at room temperature (Thermo Fisher Scientific). Optical density was measured at 450 nm using an xMark Microplate Spectrophotometer (BioRad). The area under the curve (AUC) of the OD_450_ values for all dilutions of each individual serum sample was calculated using the trapezoidal rule and expressed in arbitrary units.

#### 2.6.3. Cell-Based ELISA

MDCK cells were used to assess the expression of M2e epitopes on the surface of infected cells, which were infected with 5-fold dilutions of LAIV, LAIV/HA+4M2e or LAIV/NS+4M2e, starting with 1 MOI (number of TCID_50_ per cell). As a control of equality of MOI doses of each virus H3N2 HA-specific antibody was used.

After 24 h of incubation, plates were fixed with cold 80% acetone and incubated on ice for 20 min. Then, the plates were washed twice with PBS supplemented with 3% tween (PBST) and blocked with 50 µL of 5% non-fat dry milk in PBS at 37 °C. After 1 h incubation, plates were washed with PBST and quenched with 50 µL of 0.8 M H_2_O_2_ at room temperature for 15 min. After an additional wash, primary 14C2 (1 µg/mL) or H3N2 HA-specific (1 µg/mL) antibodies diluted in 5% non-fat dry milk in PBS were added and plates were incubated at 37 °C for 1 h, followed by washing with PBST and addition of diluted 1:3000 secondary anti-mouse IgG antibody (Abcam, UK).

Afterwards, plates were washed 3 times, and antibody binding was detected with 1-Step Ultra TMB-ELISA Substrate Solution (Thermo Fisher Scientific). Once the desired color developed (approximately 15 min), 50 µL of H_2_SO_4_ stop solution were added to each well. The absorbance was measured at 450 nm using an xMark Microplate Spectrophotometer (BioRad).

#### 2.6.4. Assessment of Cell-Mediated Immune Responses

Virus-specific and M2e-specific cellular responses were assessed by an intracellular cytokine staining (ICS) assay. For this, ferret splenocytes were isolated from small pieces of ferret spleen collected five days post-challenge using 70 µm cell strainers (Becton Dickinson, Franklin Lakes, NJ, USA). Red blood cells were lysed by an ammonium-chloride potassium lysing buffer (Thermo Fisher Scientific) and the single cell suspensions were maintained in CR-0 media (RPMI supplemented with 5 mM HEPES, 1× antibiotic-antimycotic, 50 µM β-mercaptoethanol, and 40 U/mL human IL-2). For *in vitro* stimulation, 10^6^ cells were incubated either with 1 MOI of sucrose-purified H3N2 LAIV and S.A. H1N1 virus or with 1 µg/well of 3M2e recombinant protein for 1 h, followed by addition of RPMI medium supplemented with 30% FBS to reach final serum concentration 10%. Antigen-stimulated cells were incubated at 37 °C, 5% CO_2_ for 16 h, and then 50 μL of GolgiPlug solution (Becton Dickinson, USA) was added to stop protein transport. For positive assay control, a phorbol myristate acetate (PMA) was added to some wells, and the mixture was incubated for another five hours. After stimulation, the cells were stained with ZombieAqua fixable viability dye (BioLegend, San Diego, CA, USA), APC anti-CD4 (60003-MM02-A, Sino Biological, Beijing, China), and FITC anti-CD8 (ab 210326, Abcam) antibodies for 20 min in the dark. Then, the samples were washed twice with 200 μL of a staining buffer. For intracellular staining, a Cytofix/Cytoperm kit (Becton Dickinson, USA) was used according to the manufacturer’s instructions, followed by staining lymphocytes with a phycoerythrin (PE) anti-IFNγ (ab27866, Abcam) and Pacific Blue anti-CD3 (MCA1477PB, BioRad) antibodies for another 20 min. Then, the samples were washed twice with 200 μL of a wash buffer. The cells were fixed in 100 µL of Cyto-last buffer (BioLegend) and stored in a dark cool place prior to the flow cytometric analysis. At least 100,000 events were measured using a Navios flow cytometer (Beckman Coulter, Brea, CA, USA). The data were analyzed using the FlowJo software (TriStar Inc., El Segundo, CA, USA); the proportion of antigen-specific T cells was calculated by subtracting the negative control from the IFNγ-positive T cells. The gating strategy for the ICS assay is shown in Figure S1.

#### 2.6.5. Indirect Protective Effect of Immune Sera

Indirect protective effect of antibodies in serum specimens collected on day 21 after the second vaccine dose was determined in a mouse in vivo protection model as previously described [14]. Briefly, pooled sera from each vaccine group or placebo were mixed with PBS at a 1:1 ration and then heat-inactivated at 56 °C for 1 h. Then, the treated samples were mixed with 3 50% lethal doses (LD_50_) of one of the following challenge viruses: (i) a mouse-adapted H1N1 A/California/7/2009—*Cal MA (Hu/Sw)*; (ii) a recombinant A/PR8-based virus carrying M gene of A/duck/Potsdam/1402-6/1986 (H5N2) virus—*PR8 (Av/Sw)*; (iii) a recombinant A/PR8-based virus carrying M gene of A/Aichi/2/1968 (H3N2) virus—*PR8 (Hu)*, and (iv) a recombinant A/PR8-based virus carrying artificial M gene of swine-origin lineage—*PR8 (Sw)*. The serum-virus mixtures were incubated at RT for 30 min and administered intranasally to naïve BALB/c mice in a volume of 50 μL. Protective effect of ferret sera was assessed by monitoring mouse survival rates and body weight loss during 14 days post-infection. Mice losing 30% of original body weight were humanely euthanized and recorded as dead.

#### 2.6.6. Antibodies Produced by Lymphocytes in Mediastinal Lymph Nodes

Mediastinal lymph nodes (MLN) of ferrets were collected 5 days after the challenge. Single-cell suspension was prepared using 70 µm cell strainer (Becton Dickinson) and resuspended in 2 mL of CR-10 media (RPMI media supplemented with Ab/Am and 10% of FBS). After centrifugation at 300 RCF, 7 min, 22 °C and resuspension in 1 mL of CR-10, the MLN cells were counted. The isolated MLN cells were examined for their capacity to secrete both total IgG and antigen-specific IgG antibodies.

##### Total IgG in MLN

To determine the level of total IgG secreted by MLN lymphocytes, the isolated MLN cells from each ferret were resuspended in CR-0 media at 2 million cells per ml and incubated in non-binding 96-well plates in triplicates for 6 days at 37 °C, 5% CO_2_. The supernatants were collected on 2nd, 4th and 6th days of incubation (50 µL each) and kept at −20 °C until further processing. The concentration of total IgG in the samples was determined with a two-antibody sandwich ELISA. High-sorbent 96-well plates (Greiner Bio-One) were coated with a 100 ng/well of goat anti-ferret IgG (Sigma) diluted in PBS. Plates were incubated overnight at 4 °C, washed with PBST, and blocked with 1% of BSA in PBS for 30 min at 37 °C. Collected MLN supernatants were serially diluted starting from 1/10 and added to the wells of anti-ferret IgG-coated plates. Serial dilutions of purified ferret IgG native protein standard (2 mg/mL, Antibody Research Corporation, St. Charles, MO, USA) were added to the wells of each plate to generate standard curve. After 1 h incubation at 37 °C, all plates were washed with PBST and incubated with anti-ferret IgG conjugated to horseradish peroxidase (Sigma). The chromogen was produced using 1-Step Ultra TMB-ELISA Substrate Solution (Thermo Fisher Scientific) and measured at an absorbance of 450 nm. The concentration of total IgG was expressed in µg/mL from the regression curve of the standard ferret IgG titration.

##### Antigen-Specific IgG in MLN

Standard high-binding ELISA plates were coated with 50 ng/well of either H3N2 LAIV sucrose gradient purified whole virus or 3M2e protein at 4 °C overnight. The next day, the plates were washed with PBST and blocked with 1% BSA in PBS, and 10^6^ of isolated MLN cells were added in triplicates to the ELISA plates, followed by incubation for 2 days at 37 °C, 5% CO_2_. Then, after three washes with PBST, all plates were incubated with anti-ferret IgG antibody conjugated to horseradish peroxidase (Sigma). Antibody binding was detected with 1-Step Ultra TMB-ELISA Substrate Solution (Thermo Fisher Scientific) after incubation for 15 min at room temperature and stopping with H_2_SO_4_. Optical density was measured at 450 nm using an xMark Microplate Spectrophotometer (BioRad).

### 2.7. Assessment of Protection against Heterologous Virus Challenge

Groups of four ferrets immunized with each tested vaccine or placebo were challenged with 10^6^ log_10_EID_50_ of heterologous wild-type A/South Africa/3626/2013 (H1N1) virus on day 21 after the second vaccine dose. The virus was administered intranasally to anesthetized ferrets in a volume of 0.5 mL. The challenge phase lasted for five days, when the animals were euthanized for blood and tissue collection for further immunological, virological and/or histopathological analyses.

#### 2.7.1. Protection by Clinical Outcomes

Body weight and clinical symptoms of disease (sneezing, nasal discharge and reduction in overall animal activity) were daily recorded for each animal. Temperature increase over the challenge phase was monitored by implanted temperature loggers that recorded body temperature every 30 min. The data from the temperature loggers was retrieved during the autopsy on day 47 of the experiment, and the temperature increase was calculated as an area under the curve (AUC) of the temperature data relative to the basal temperature previously determined for each animal (AUC delta T parameter). Body weight loss (% decrease relative to the weight on day 42) was also analyzed by the AUC parameter. In both cases the AUC data was calculated by the trapezoidal rule.

#### 2.7.2. Protection by Virological Outcomes

Nasal wash specimens were collected daily until day five post challenge. Infectious viral titers were determined by end-point titration in eggs as described above and expressed as log_10_EID_50_/mL. In addition, viral titers were determined in nasal turbinates, trachea and lungs collected on day 5 after challenge. The lung tissues of each animal were harvested from all five lung lobes in separate 2 mL round-bottom tubes. Tissue samples were weighed and stored frozen at −70 °C until further processing. 1 mL of sterile cold PBS was added to each tissue sample and tissue homogenates were prepared using a small bead mill (TissueLyser LT, Qiagen, Hilden, Germany). Viral titers in the supernatants were determined by titration in eggs as described above and expressed as log_10_EID_50_/gram tissue.

#### 2.7.3. Protection by Histopathological Outcomes

For histopathological evaluation of the damage to the lower respiratory tract, small pieces were excised from four lung lobes of each sacrificed animal and were placed in a 10% neutral buffered formalin solution for fixation. Then the samples were cut and placed into cassettes, followed by an automatic processing using a Tissue-Tek VP1 automated histoprocessor (Sakura). The resulting paraffin blocks were cut into sections with a thickness of 3 μm, which, after drying, were stained with hematoxylin and eosin. Histological preparations were examined under a Leica DM1000 microscope at 400× magnification. For each lung lobe, two sections were placed in a glass slide and all samples (32 per study group, 128 in total) were analyzed by a blinded histologist. The decrease in the airiness of the pulmonary lobes’ parenchyma was treated as an affective tissue. The area of affected lung tissue in % of all field area was measures by a microscope at 200× magnification with a calibrated objectives micrometer taking into account 15–40% compression of wet tissues due to fixation and sample histoprocessing [15]. Histological evaluation of tissue injury also included the assessment of alveolar septum thickening by a microscope with calibrated objectives [16].

### 2.8. Statistical Analyses

Data were analyzed with the statistical module of Graph Pad Prism 6 software (GraphPad Software, San Diego, CA, USA). Statistically significant differences between study groups were determined by ANOVA with Tukey’s multiple comparison test (in vitro and in vivo studies). Differences in the survival rates after challenge were analyzed by a log-rank Mantel–Cox test. *p* values of <0.05 were considered significant.

## 3. Results

### 3.1. Generation and In Vitro Characterization of Recombinant LAIV+4M2e Viruses

**Figure 2:**
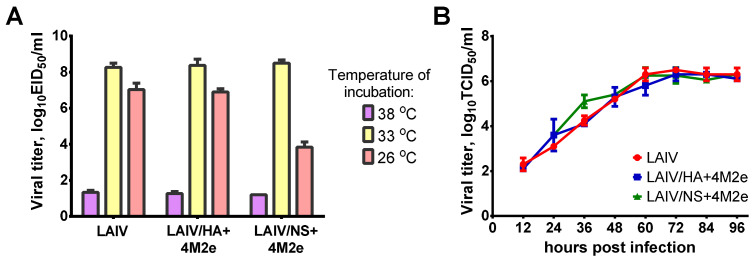
Growth characteristics of LAIV viruses used in this study. (**A**) Infectious viral titers in eggs incubated at different temperatures. (**B**) Kinetics of viral growth in MDCK cells at optimal temperature 33 °C and multiplicity of infection 0.01.

In an early proof-of-concept study we demonstrated the feasibility of an approach to enhance M2e-specific antibody responses after vaccination. The LAIV viruses expressing four M2e tandem repeats from the N-terminus of HA1 subunit induced significantly higher levels of M2e-specific antibody than classical LAIVs which resulted in improved cross-protection of immunized mice against heterosubtypic influenza viruses [6,7]. However, the inserted consensus M2e epitopes of human, swine and avian influenza viruses were designed based on previous publications and consensus sequences reported prior to the 2009 pandemic [17]. We previously conducted in-deep analysis of conservation of M2e sequences among all influenza A viruses circulating in human, avian and swine reservoirs and suggested new consensus M2e sequences to better reflect most of circulating viruses [5]. We explored two ways of inserting this new 4M2e construct into genome of H3N2 LAIV virus: (i) between the signal peptide and the N-terminus of HA1 subunit (LAIV/HA+4M2e), and (ii) to the C-terminus of NS1 protein truncated to 126 amino acids, via the P2A self-cleavage site (LAIV/NS+4M2e) (Figure 1A). Both recombinant viruses grew efficiently in eggs and MDCK cells at optimal temperature 33 °C (Figure 2). Furthermore, all viruses possessed temperature-sensitive phenotype as very limited replication occurred in eggs at 38 °C. Interestingly, the cold-adapted phenotype was preserved in LAIV/HA+4M2e virus, whereas the ability to grow at low temperature was impaired in the LAIV/NS+4M2e candidate, indicating the impact of modified NS1 protein on virus growth properties at lower temperatures (Figure 2A). Nevertheless, the kinetics of viral replication on MDCK cells was identical for all LAIV viruses used in the study, suggesting that the insertion of the 4M2e cassette did not affect viral replication in mammalian cells at optimal conditions (Figure 2B).

**Figure 3:**
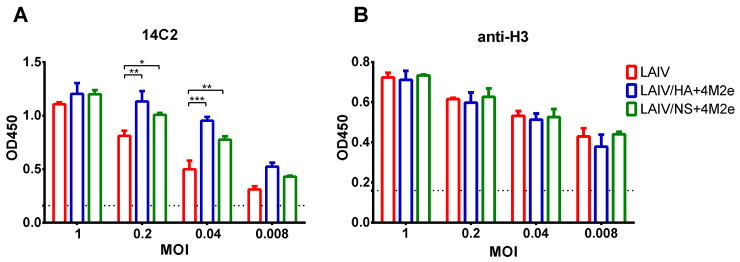
Detection of M2e antigen in infected cells. MDCK cells were infected with various MOIs of the studied viruses and were fixed 24 hpi. Detection of M2e protein was performed using 14C2 monoclonal antibody (**A**) Detection of H3 protein was performed using H3N2 HA-specific monoclonal antibody (**B**) Dashed lines indicate the mean OD_450_ values of the uninfected control wells. Data were analyzed by two-way ANOVA with Tukey’s post-hoc multiple analyses test. * *p* < 0.05; ** *p* < 0.01; *** *p* < 0.001.

Expression of M2e epitopes by the recombinant LAIV viruses was assessed by infecting MDCK monolayers with these viruses at different MOIs, followed by detection of M2e antigen by the M2e-specific monoclonal antibody 14C2. Classical H3N2 LAIV was used as a control virus. It is known that M2 protein is abundantly present on the surface of virus-infected cells, therefore significant levels of 14C2 antibody binding were detected in cells infected with control LAIV virus. Nevertheless, the incorporation of additional M2e epitopes into HA or NS1 molecule of the vaccine virus resulted in stronger binding of the antibody to the cells infected with the same MOIs as the control virus, indicating that higher quantities of M2e epitopes are present within the infected cells (Figure 3A). To confirm that this stronger binding of 14C2 antibody results from enhanced expression of M2e proteins by the recombinant LAIV+4M2e viruses rather than by the better viral growth properties, we also performed cell ELISA using 4F7 monoclonal antibody which binds HA molecule of H3N2 viruses. As expected, all three LAIV viruses had similar levels of HA proteins on the infected cells at all MOIs tested, suggesting that there was now impairment of viral replication when the genome of LAIV virus was modified (Figure 3B).

### 3.2. Virus Replication in Ferrets’ Upper Respiratory Tract

**Figure 4:**
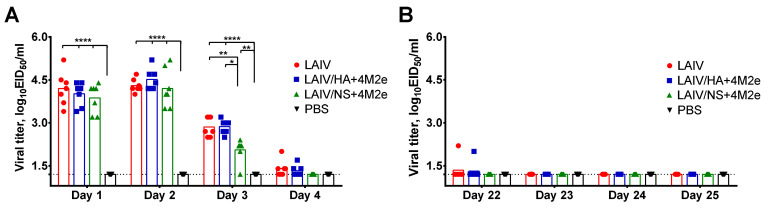
Shedding of LAIV viruses after intranasal immunization of ferrets. Groups of seven animals were inoculated i.n. with indicated viruses or placebo (PBS) at a dose of 10^7^ EID_50_, in the volume 500 µL. The second dose was administered 21 days later. Shedding of LAIV viruses was determined by titration in eggs of nasal wash samples collected during 4 days after the first (**A**) and the second (**B**) vaccinations. Data were analyzed by two-way ANOVA with Tukey’s post-hoc multiple analyses test. * *p* < 0.05; ** *p* < 0.01; **** *p* < 0.0001.

Groups of seven ferrets were intranasally immunized with two recombinant viruses, as well as with the H3N2 LAIV control virus, at a dose 10^7^ EID_50_ per animal, at a two-dose regimen with 21 days interval. All three LAIV viruses replicated efficiently in ferrets’ upper respiratory tract after the first vaccine dose, as was determined by titration of nasal wash specimens in eggs. Interestingly, during first two days the tree viruses had similar levels of virus shedding, whereas at day 3 the LAIV/NS+4M2e candidate showed significantly lower titers, compared to the other two vaccine viruses, suggesting the impact of modified NS gene on the rate of virus clearing from the ferrets URT (Figure 4A). All viruses were almost cleared by day 4 post inoculation. There was no virus replication after the second administration of the LAIVs, suggesting that the induced anti-influenza immunity prevented active viral replication in the respiratory tissues of the animals (Figure 4B). Overall, the insertion of 4M2e cassette into LAIV genome did not alter the ability of vaccine viruses to replicate in the ferrets’ upper respiratory tract, indicating their immunogenic potential.

### 3.3. Safety of the LAIV+4M2e Vaccine Candidates

**Figure 5:**
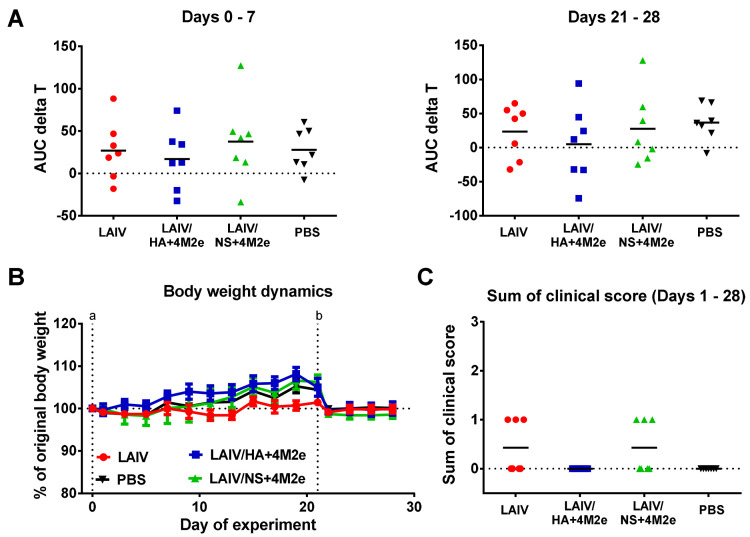
Safety of the LAIV candidates in ferrets as measured by clinical outcomes. (**A**) Area under the curve (AUC) of delta T values for individual ferrets during one week after the first vaccine dose (left graph) and during one week after the second vaccine dose (right graph). (**B**) Ferrets’ body weight dynamics after the first (a) and the second (b) dose of the vaccines or placebo. (**C**) Sum of clinical scores for each animal during 28 days of the experiment.

Safety assessment of the new recombinant vaccine viruses in the ferret model was conducted by monitoring body weight loss and overall clinical signs of infection during the course of immunization, until day 7 after the second dose. Importantly, body temperature was recorded every 30 min using implanted temperature loggers, which allows measurement of fever by the area under the curve (AUC) of delta T parameter (temperature increase/decrease relative to the background mean T calculated individually for each ferret). All vaccine candidates were safe and well-tolerated, as no significant temperature increase was detected in any of the LAIV group relative to placebo (PBS), neither after the first, nor the second intranasal inoculation (Figure 5A). Furthermore, no significant differences between groups were seen in the dynamics of ferrets’ body weight. There was some decrease in body weight after the second vaccine dose, but this decrease was seen for all study groups, most probably due to the bleeding performed to assess vaccines immunogenicity (Figure 5B). The safety of the vaccines was also confirmed by the absence of significant clinical signs of infection in any of the study group (Figure 5C). Finally, during histopathological evaluation of respiratory tissues collected 7 days after the second dose of any LAIV, neither acute (inflammatory, dystrophic and destructive) nor chronic (atrophic and sclerotic) changes in the parenchymal organs and stromal component were found (Figure S2), further indicating that the recombinant LAIV viruses were safe when tested in a ferret model.

### 3.4. Antibody Immune Responses after Vaccination

**Figure 6:**
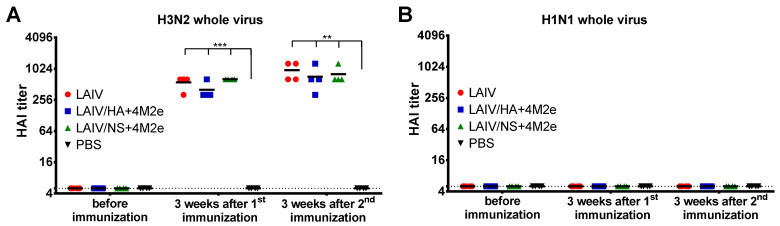
Hemagglutination inhibiting (HAI) antibody titers in ferrets immunized with studied LAIV viruses. Ferret sera were collected three weeks after the first and the second intranasal immunization. The antibody titers were assessed in HAI assay using the H3N2 LAIV (**A**) or S.A. H1N1 (**B**) whole virus as an antigen. Data were analyzed by two-way ANOVA with Tukey’s post-hoc multiple analyses test. ** *p* < 0.01; *** *p* < 0.001.

**Figure 7:**
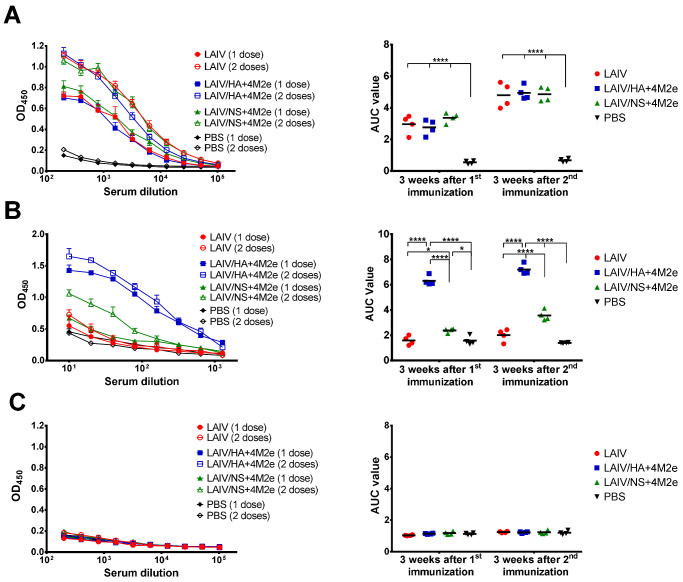
Serum IgG antibody immune responses after vaccination. Ferret sera were collected three weeks after the first and the second intranasal immunization. IgG antibody levels were assessed in ELISA with different antigens. (**A**) H3N2 LAIV whole virus. (**B**) 3M2e recombinant protein. (**C**) S.A. H1N1 whole virus. Left panel shows mean OD_450_ values for each serum dilution. Right panel shows the area under the OD_450_ curve values for each individual animal. Data were analyzed by two-way ANOVA with Tukey’s post-hoc multiple analyses test. * *p* < 0.05; **** *p* < 0.0001.

Antibody immune responses to i.n. immunization with the LAIV viruses were measured in ferret sera 21 days after each immunization. HAI antibody titers to the homologous H3N2 LAIV virus were raised to significant levels already after a single vaccine dose, and were within one 2-fold dilution between the three LAIV test groups (Figure 6A). The second dose did not significantly increase the H3-specific HAI titers, most probably due to the lack of viral replication after the second inoculation (Figure 4B). Nevertheless, there was significant boost of H3N2 whole virus-specific IgG antibody with the second immunization for all three LAIVs tested, suggesting that the booster vaccine dose indeed was required to induce robust virus-specific antibody responses (Figure 7A).

Similar to HAI titers, there was no difference between the investigated vaccines in terms of induction of H3N2 virus-specific IgG antibodies either after the first or after the second dose (Figure 7A). Most importantly, when the ELISA plates were coated with the 3M2e recombinant protein, significant IgG antibody levels were detected only in ferrets immunized with the LAIV/HA+4M2e and LAIV/NS+4M2e, but not the classical LAIV virus (Figure 7B). Interestingly, the M2e-specific antibody levels were much higher after immunization with recombinant LAIV virus expressing the 4M2e cassette from the HA molecule, than the virus with modified NS1 gene. Strikingly, the robust M2e antibody responses were detected after a single immunization with the LAIV/HA+4M2e strain with almost no booster effect of the second dose, whereas the second dose of the LAIV/NS+4M2e virus was required to raise substantial M2e-specific IgG antibody titers (Figure 7B). These data suggest that the presence of the additional M2e copies on the surface of viral particles results in better immunogenicity of these small proteins, compared to their expression from the NS1 open reading frame inside an infected cell.

As expected, no significant H1N1-specific HAI and IgG antibody titers were detected in ferrets immunized with classical or recombinant H3N2 LAIV viruses (Figure 6B and Figure 7C).

We further assessed the binding capacity of vaccine-induced serum IgG antibodies in a cell-based ELISA assay. For this, MDCK cells were infected with a panel of PR8-backbone influenza viruses that contain M genes of different origin, with M2e sequences perfectly matching the four M2e sequences contained in the 4M2e cassette (Figure 1A). Since M2 proteins, along with HA and NA, are abundantly expressed on the surface of virus-infected cells, these cells should also capture the M2e-specific antibody present in the sera of vaccinated animals, not only the HA- and NA-specific cross-subtype specific antibody. Indeed, the cells infected with all four viruses could bind significantly higher quantities of IgG antibody from the serum samples of ferrets immunized with both recombinant LAIV-4M2e viruses, compared to the sera of LAIV-immunized animals, suggesting that the pool of M2e-targeted antibodies raised by the universal LAIV candidates can efficiently recognize virus-infected cells and have a capacity to improve protective potential of the traditional LAIV (Figure S3).

### 3.5. Protection against Heterologous H1N1 Influenza Virus

To assess the capacity of the new vaccine candidates to protect against heterosubtypic influenza virus, immunized ferrets were challenged on day 42 of the study with A/South Africa/3626/2013 (pH1N1) virus at a dose 10^6^ EID_50_ per animal, which is known to cause severe disease in this animal model when administered at a such high dose [18]. Protective effect was assessed by virological, clinical and histopathological endpoints.

#### 3.5.1. Titers of H1N1 Challenge Virus in the Upper and Lower Respiratory Tract

**Figure 8:**
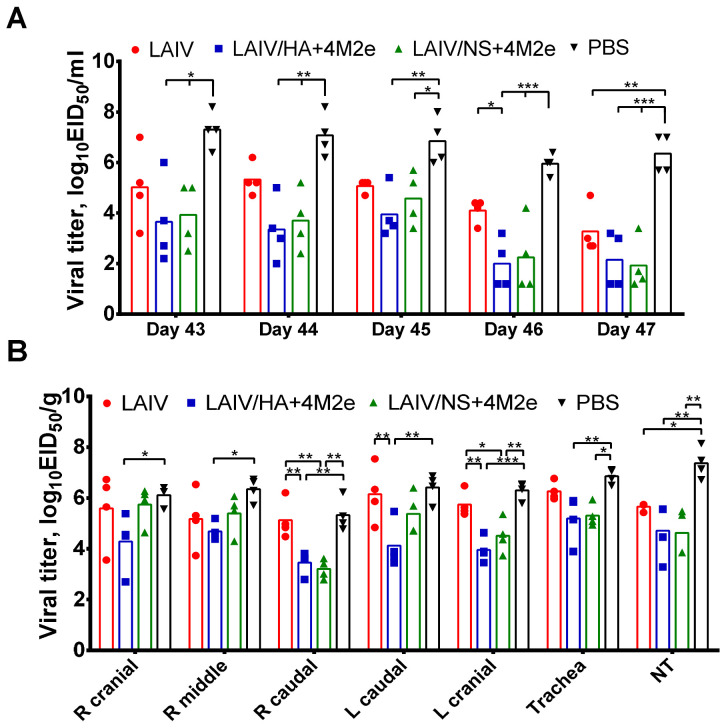
Replication of H1N1 challenge virus in the respiratory tract of immunized ferrets. Groups of four ferrets were infected i.n. with 10^6^ log_10_EID_50_ of S.A. H1N1 challenge virus three weeks after the second vaccine dose. (**A**) Nasal wash specimens were collected daily after the challenge and infectious viral titers were determined in eggs and expressed as log_10_EID_50_/mL. (**B**) Small pieces of tissues (nasal turbinates, trachea and lungs) were collected from euthanized ferrets five days post-challenge. Lung tissues were collected from five lung lobes separately for each animal. Viral titers in tissue homogenates were determined by titration in eggs and expressed in log_10_EID_50_/gram tissue. Data were analyzed by two-way ANOVA with Tukey’s post-hoc multiple analyses test. * *p* < 0.05; ** *p* < 0.01, *** *p* < 0.001.

Nasal wash specimens of ferrets were collected daily after challenge until day 47 of the study, and infectious virus titers were determined by endpoint titration in eggs. Mock-immunized animals shed the virus at high level during all challenge phase reaching the mean group titers 6.0–7.3 log_10_EID_50_/mL (Figure 8A). 

Ferrets immunized with classical H3N2 LAIV virus had slightly reduced mean titers compared to the PBS group at all days, however statistical significance was reached only on day 5 post-challenge, suggesting that the induced immunity was not sufficient for fast clearance of the heterologous challenge virus from the URT. In contrast, ferrets immunized with both LAIVs expressing 4M2e cassettes had significantly reduced NW viral titers at all time points, with two out of four animals having the titers below the limit of detection on day 4 after challenge (Figure 8A). In concordance with the NW titers, all three vaccine groups had significantly reduced titers in the nasal turbinates of the ferrets on day 5 post-challenge, suggesting that even classical LAIV can reduce virus titer in the URT, although the speed of virus clearance was clearly dependent on the presence of additional M2e epitopes in the vaccine virus (Figure 8B). Strikingly, there was no reduction of virus titers in trachea or lung tissues in the LAIV-immunized ferrets, compared to the mock-immunized animals (Figure 8B), whereas ferrets from both LAIV+4M2e groups showed virus reduction in trachea and lungs. Interestingly, the LAIV/HA+4M2e vaccine demonstrated reduction in virus pulmonary titers for all five lung lobes tested, whereas the LAIV/NS+4M2e-immunized animals had significantly reduced titers only in two out of five lung lobes, suggesting that the HA-modified vaccine produced more potent cross-protective immunity than the NS-modified candidate (Figure 8B). Overall, although the induced heterosubtypic immunity was not sterile for any of the studied vaccines, the incorporation of the four M2e epitopes into genome of H3N2 LAIVs significantly improved virological outcomes of a high-dose heterosubtypic influenza virus challenge.

#### 3.5.2. Clinical Evaluation of H1N1 Challenge Virus-Induced Pathology

**Figure 9:**
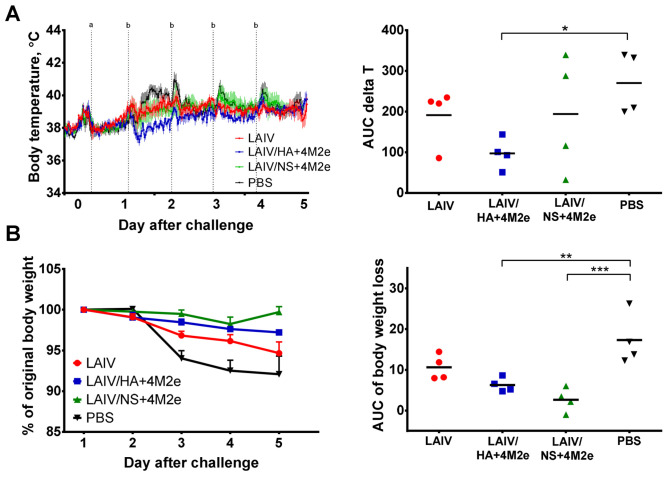
Protection against challenge virus by clinical outcomes. (**A**) Data of body temperature recorded every 30 min were retrieved from the implanted temperature loggers (left graph). The AUC of delta T values were determined for each ferret based on an individual basal temperature, using the trapezoidal rule (right graph). (**B**) Body weigh was recorded daily over the challenge phase and is presented as the mean % of original body weight of each test group with SEM (left graph). The AUC values for the % weight loss were calculated for each animal using the trapezoidal rule (right graph). Data were analyzed by one-way ANOVA with Tukey’s post-hoc multiple analyses test. * *p* < 0.05; ** *p* < 0.01, *** *p* < 0.001.

Ferrets from all test groups experienced some elevations in body temperature during the challenge phase. Analysis of the dynamics of body temperature change for each ferret by determining the area under the curve of delta T parameter revealed significant reduction in the AUC values for the LAIV/HA+4M2e group, compared to the mock-immunized ferrets (Figure 9A). 

Although one out of four and two out of four ferrets in the LAIV and LAIV/NS+4M2e vaccine groups, respectively, demonstrated reduced AUC delta T values, the difference in the mean AUC values relative to the PBS group did not reach statistical significance, most probably due to the low number of animals in each group. Interestingly, all three vaccine candidates showed the improvements in the body weight loss parameter if compared to the PBS group, although statistically significant differences in the AUC of body weight loss values were found only for the two recombinant LAIV+4M2e vaccines, but not for the LAIV control virus (Figure 8B).

#### 3.5.3. Histopathological Evaluation of H1N1 Challenge Virus-Induced Pathology

We additionally analyzed the tissue damage of trachea and lung specimens on a microscopic level. Since challenge virus was detected in the lung tissues of all ferrets, pathological changes were observed in all tissue specimens, however the extent of the damage differed between the test groups. The most significant pathological changes were found in the PBS and LAIV groups, with extensive foci of serous-hemorrhagic pneumonia, as well as emphysematous enlarged areas (Figure S4A). The ciliated epithelium of the bronchi contained large light nuclei and vacuolated cytoplasm with basophilic inclusions. In most of the analyzed slides from both control groups, the alveolar spaces were filled with fresh fibrinous effusion, which also included erythrocytes, necrotic alveolocytes, as well as diffuse polymorphic cell infiltration. The vascular bed was full-blooded, with sludge in the lumen of the vessels, and areas of non-organized parietal fibrin deposition. Destructive processes of the infection in the PBS and LAIV groups were also represented by necrotizing alveolitis and destructive bronchiolitis. In contrast, pathological changes of the lung tissues if ferrets immunized with either LAIV+4M2e vaccine were presented only by local foci of serous-hemorrhagic pneumonia and distelectasis of the pulmonary parenchyma (Figure S4A). There was no significant difference in pathological changes of trachea tissues between the study groups.

Analysis of the sum of pathomorphological scores did not reveal significant differences between the groups due to the high intragroup variance of lesions in different lobes of the lungs (*p* = 0.099, data not shown). However, morphometric assessment of alveolar septum thickness showed statistically significant differences between the PBS vs. LAIV (*p* = 0.0205), PBS vs. LAIV/HA+4M2e (*p* < 0.0001) and PBS vs. LAIV/NS+4M2e (*p* = 0.0481) (Figure S4B). Semi-quantitative assessment of the area of affective tissue in the preparations also revealed differences between the PBS vs. LAIV (*p* = 0.0002) and PBS vs. LAIV/HA+4M2e (*p* = 0.0279) (Figure S4C).

### 3.6. Mechanisms of Improved Protection Afforded by LAIV+4M2e Vaccine Candidates

We conducted a deeper analysis of effector immune responses following virus challenge to find some immunological correlates of improved cross-protection provided by the recombinant LAIVs carrying additional M2e epitopes. We collected splenic tissues from ferrets five days after challenge and assessed the proportions of antigen-specific T cells in an ICS assay after in vitro stimulation of splenocytes with H3N2 LAIV and S.A. H1N1 whole viruses or with 3M2e recombinant protein. The proportions of IFNγ-secreting cells varied significantly between ferrets within a group, suggesting the impact of heterogeneous genetic background of the outbred animals (Figure S5). Nevertheless, activated CD4 and CD8 T cells were found in all study groups after stimulation with whole viruses, while no ferret showed an increase in M2e-specific T cells, suggesting that the M2e protein-specific cellular responses were not the main driver of the observed cross-protection.

**Figure 10:**
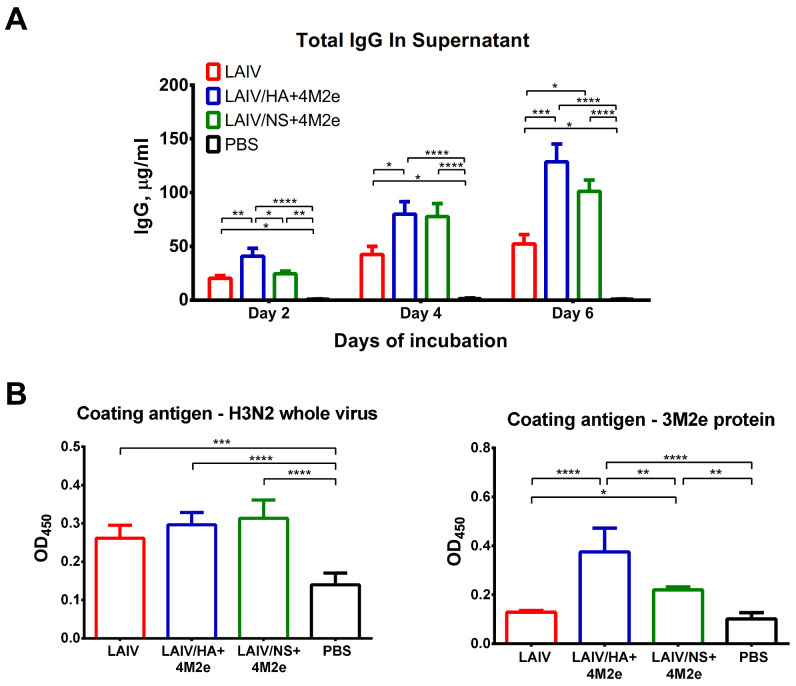
Antibodies produced by MLN cells. (**A**) Mediastinal lymph nodes were collected 5 days post-challenge and single-cell suspensions were incubated in culture medium without addition of any antigen. Supernatants were collected on days 2, 4 and 6 and the level of total IgG was determined by ELISA from the regression curve of the standard ferret IgG titration. (**B**) The same isolated MLN cells were cultured in ELISA plates coated with indicated antigens, followed by development with secondary anti-ferret IgG antibodies and chromogenic substrate. Data were analyzed by two-way (**A**) or one-way (**B**) ANOVA with Tukey’s post-hoc multiple analyses test. * *p* < 0.05; ** *p* < 0.01, *** *p* < 0.001, **** *p* < 0.0001.

More likely, the improved outcomes of the H1N1 virus infection in ferrets from the LAIV/HA+4M2e and LAIV/NS+4M2e test groups, compared to PBS and LAIV groups, was due to the induction of robust cross-reactive M2e-specific serum IgG antibody (Figure 7 and Figure S2). Indeed, there was more pronounced secretion of IgG antibodies by MLN cells collected from LAIV/HA+4M2e and LAIV/NS+4M2e-immunized ferrets on day 5 after H1N1 virus challenge, compared to the LAIV- and mock-vaccinated animals (Figure 10A). Furthermore, in vitro stimulation of these MLN cells revealed that the M2e-specific antibody most probably contributed to these elevated IgG antibody levels (Figure 10B). Consistent with the levels of serum IgG antibody post-vaccination (Figure 7), the magnitude of M2e-binding antibody responses was significantly higher in the LAIV/HA+4M2e than in the LAIV/NS+4M2e study group 5 days after challenge, suggesting that M2e-specific memory B cells are readily activated upon subsequent heterologous virus infection and the produced antigen-specific antibodies are capable of mediating cross-protection (Figure 10B).

To find possible immunological correlates of protection against the heterologous challenge virus we performed correlation analyses between various immunological outcomes and the infectious titers of the H1N1 challenge virus in the ferrets’ respiratory tissues (the lung titers were calculated as the mean titer of all five lung lobes for each animal). There was no statistically significant dependence of the pulmonary virus replication and the levels of H3N2 whole virus-specific serum IgG antibody measured on day 42 of the study (Figure S6A, Spearman r = −0.4265, *p* = 0.1011). Similarly, weak correlation was observed for these antibodies and viral titers in trachea (r = −0.4797, *p* = 0.0624). Noteworthy, the virus-specific IgG antibodies correlated well with viral activity in the nasal turbinates (r = −0.7235, *p* = 0.0021). Although we did not measure the levels of virus-specific mucosal IgA levels, it can be assumed that all three LAIVs may have elicited similar IgA responses, which, due to their cross-reactive nature, may have cleared the virus from the URT. Nevertheless, the strongest association of the reduction in virus pulmonary titers was found for the levels of M2e-binding serum IgG antibody post-vaccination (Figure S6B, r = −0.7838, *p* = 0.0003), M2e-specific antibody secreted by the MLN cells collected 5 days post-challenge (Figure S6C, r = −0.8344, *p* = 0.0001), as well as total IgG concentration secreted by these MLN cells in vitro (Figure S6D, r = −0.7853, *p* = 0.0005). The three latter immunological outcomes also correlated well with viral loads in trachea and nasal turbinates, suggesting that the M2e-specific antibodies were the main drivers of improved cross-protection afforded by LAIV+4M2e vaccine candidates, compared to the control LAIV strain.

**Figure 11:**
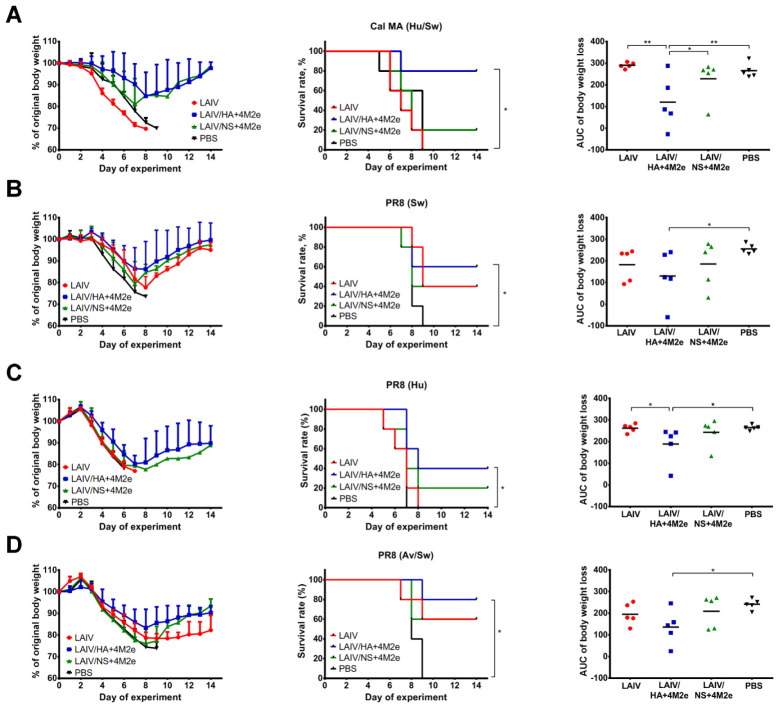
Protective effect of ferret immune sera in a mouse in vivo protection assay. Pooled sera collected from immunized ferrets three weeks after the second dose was mixed with one of the four viruses at a dose of 3 LD50 and inoculated intranasally to naïve C57BL/6J mice (n = 5). Survival rates and weight loss were monitored during 14 days after infection. (**A**) Cal MA H1N1 (M2e of human/swine lineage). (**B**) A/PR8 virus carrying engineered M gene of swine lineage. (**C**) A/PR8 virus carrying M gene of A/Aichi/2/1968 (H3N2) (M2 of human lineage). (**D**) A/PR8 virus with M gene of A/duck/Potsdam/1402-6/1986 (H5N2) virus (M2e of avian/swine lineage). Data were analyzed by log-rank Mantel-Cox test (middle panel) or one-way ANOVA with Tukey’s post-hoc multiple analyses test (right panel). * *p* < 0.05; ** *p* < 0.01.

To further confirm that the recombinant LAIV+4M2e-induced M2e-specific antibody mediated the improved protection we tested the ferret immune sera collected 3 weeks after the second vaccine dose in a mouse in vivo protection model. To this end, in addition to a lethal mouse-adapted Cal/09 H1N1 virus (representing human/swine M2 lineage), a panel of PR8-based pathogenic viruses was used to cover the remaining three M2e epitopes that were inserted into genome of the H3N2 LAIV virus. LD_50_ doses of each virus have been previously determined for the C57BL/6J mouse strain (data not shown), and the dose of 3 LD_50_ of each virus was used for the assessment of an indirect protective effect of ferret sera. Strikingly, significant improvements in mouse weight loss and survival rates for all four challenge viruses were observed only for the sera derived from ferrets immunized with LAIV/HA+4M2e vaccine, but not for the LAIV/NS+4M2e candidate (Figure 11). Although the higher proportion of challenged mice survived after passive transfer of sera from LAIV/NS+4M2e-immunized ferrets, there was still 20–60% lethality for different challenge viruses, thus resulting in the scattered AUC of weight loss values. Nevertheless, comparing the mean AUC values between groups found statistically significant differences between the LAIV/NS+4M2e and PBS group for all four viruses tested. Since all vaccine candidates used in this study had comparable H3N2 virus-specific serum antibodies and very low cross-reactive H1N1-specific antibody were found, it is highly probable that the magnitude of M2e-specific antibody response has played a critical role in the antibody-mediated heterosubtypic protection.

## 4. Discussion

Traditional influenza vaccines have been shown to substantially reduce the burden of influenza among various populations [19]. However, these vaccines mainly induce neutralizing antibodies to viral epitopes that are mutation-prone, and the virus easily escapes the vaccine-induced immunity. To overcome the issue of constant antigenic drift, the composition of influenza vaccines needs to be updated frequently, and annual vaccination is recommended to maintain a sufficient level of immunity. However, a recent systematic review and meta-analysis revealed the negative impact of repeated vaccination on the effectiveness of licensed influenza vaccines [20]. Furthermore, a wide range of natural hosts and different mutation pathways do not yet allow predicting a future circulating strain, despite significant progress in this area [21]. All these problems explain the need to develop universal influenza vaccines that would induce broadly reactive and durable immunity, thereby protecting vaccinated people against the evolved seasonal influenza viruses, as well as against new pandemic variants [22].

A variety of strategies have been explored to redirect the vaccine-induced immune responses from variable to more conserved protein targets, and the M2e epitope is one of the most commonly used target antigens in these studies. To increase the immunogenicity of this small protein, different carriers are being used, and several successful prototypes have reached clinical phase of development (reviewed in [5,23,24]). In our early proof-of-concept study, we demonstrated that cold-adapted Leningrad/17-based LAIV viruses can serve as viral vectors to deliver tandem M2e repeats to target cells and stimulate the induction of high levels of M2e-specific antibodies that improve cross-protective potential of the vaccine [6,7]. In general, most of the studies of M2e-based universal vaccine candidates are limited to their assessment in a mouse model of infection. There are only a few published studies where such vaccines were assessed in other animals (ferrets, monkeys, pigs, dogs) [25,26,27,28]. In the current study, we evaluated two universal LAIV prototypes in ferrets, genetically outbred animals, which are considered the best test system for studying influenza in humans, since they have distinct patterns of T-cell reactivity as a result of heterogeneity at the MHC locus, and are also susceptible to infection with virulent influenza viruses with similar to humans manifestation of the disease [8].

The two universal LAIV+4M2e candidates differed by the site of insertion of the 4M2e cassette. The first recombinant virus expressed the 4M2e foreign antigen from the N-terminus of the H3 HA1 subunit, similar to the previously reported H1N1 and H7N9 LAIV+4M2e candidates studied in mice [6,7]. Since this strategy will require regular update of the corresponding HA molecule to match the antigenic properties of circulating seasonal influenza viruses, we created another recombinant virus in which the 4M2e cassette is encoded in the open reading frame of the NS1 protein, which, in turn has been truncated to 126 residues. Since the NS gene originates from the Leningrad/17 master donor virus, there is no need to update this gene when seasonal viruses evolve. Here, the transgene is not present in the viral particle, but should be translated within the infected cell, because the NS1 protein is abundantly expressed in the early stages of infection [29]. Such modification of LAIV genome has been previously shown as a promising strategy for the induction of robust cytotoxic T-cell responses to the inserted immunodominant CD8 T-cell epitope of a foreign pathogen [30,31,32]. In vitro studies confirmed that regardless of how the 4M2e cassette was inserted into the LAIV genome, M2e proteins were expressed at significantly higher levels when mammalian cells were infected with the LAIV+4M2e recombinant candidates than with the classic LAIV strain, demonstrating the LAIV’s ability to properly deliver additional M2e antigens to target cells. It is important that the inserted 4M2e cassette did not disturb the replicative properties of the LAIV virus: both chimeric vaccines grew to comparable titers in eggs and MDCK cells, as well as in the upper respiratory tract of ferrets. Of note, our previous studies of NS1-modified recombinant LAIV viruses carrying immunogenic epitopes of other respiratory viruses demonstrated their inability to replicate in the mouse respiratory tract, suggesting that modulation of the LAIV virus growth properties by modification of NS1 protein is host-specific [31,32]. Importantly, all LAIVs studied in this experiment were safe and did not induce any infection-related pathology in immunized ferrets.

As expected, active replication of all three LAIV viruses promoted the development of a potent humoral immune response to the whole virus antigen, with significant boosting effect of the second dose. Importantly, there was no reduction in homologous HAI or serum IgG titers in ferrets immunized with recombinant LAIV+4M2e candidates, relative to the traditional LAIV virus, suggesting that none of the genetic modifications had an impact of influenza virus-specific humoral immunity. In line with previously published data, immunization with unmodified influenza virus (i.e., H3N2 LAIV) did not elicit notable levels of M2e-binding antibodies in ferrets [33]. However, the insertion of the 4M2e cassette into LAIV genome significantly increased the M2e-specific antibody levels, with the LAIV/HA+4M2e variant being remarkably more immunogenic than the LAIV/NS+4M2e counterpart. Moreover, a single dose of the HA-modified vaccine candidate was sufficient for eliciting powerful M2e response, whereas at least two doses of the NS1-modified vaccine was required for induction of significant M2e-specific antibody levels. These data indicate that distinct modes of expression of the 4M2e insert from the two viral proteins result in different pathways for antigen presentation, thus resulting in such a divergent magnitude of the response. To date, a fairly large amount of data has been accumulated regarding the development of viral-vectored vaccines based on the influenza virus, where the transgene was inserted into the NS1 open reading frame. In most strategies, the gene of interest is either fused to a truncated NS1 protein or inserted via a stop-start cassette, and only a few studies report transgene insertion using the 2A auto-cleavage site (reviewed in [34]). However, whether the insertion of 4M2e cassette into NS1 using a different cloning strategy will improve the immunogenicity of the M2e epitopes is unknown and requires further research.

The main objective of this study was the assessment of cross-protective potential of the new universal influenza vaccine candidates in the ferret model. For this purpose, the immunized ferrets were challenged with the S.A. H1N1 virus, which is known to cause significant pathology in this animal model [18,35]. Unlike H3N2 vaccine viruses, this subtype belongs to the Group 1 hemagglutinin, and no cross-reactive IgG responses to the challenge virus were found in ferret immune sera. It is known that classic LAIVs have a potential to protect against heterologous influenza viruses due to the induction of T-cell immune responses, as well as mucosal IgA antibody [36,37,38]. Indeed, on day 5 post-challenge, there was a decrease in upper respiratory viral load in the LAIV group compared to the PBS group. In addition, LAIV-immunized ferrets showed less severe signs of lung pathology than control animals. However, the LAIV itself was unable to protect ferrets against H1N1 virus pulmonary replication and body weight loss, whereas both recombinant LAIV+4M2e vaccines induced sufficient immunity to reduce viral titers in all tested respiratory tissues.

Importantly, since the two universal LAIV candidates induced comparable humoral responses to whole influenza virus but were differently immunogenic in terms of inducing M2e-specific antibodies, it became possible to investigate the cross-protective potential of the M2e antibodies. Thus, ferrets immunized with LAIV/HA+4M2e were better protected against viral replication and clinical manifestation of the disease than the LAIV/NS+4M2e-vaccinated animals. Subsequent analysis revealed a strong correlation between virus titers in the respiratory tract and the M2e antibody levels, but not H3N2, prior to challenge. Furthermore, the protection was strongly associated with the levels of M2e-specific antibodies secreted by the MLN cells five days post-challenge. These data suggest that protection against a heterologous influenza virus infection was mediated by recall M2e-specific IgG responses in draining lymph nodes. Similar results were observed in a mouse model, where the improved cross-protection afforded by a recombinant H3N2 influenza virus expressing the 4M2e cassette in a chimeric hemagglutinin was associated with significant increases of M2e specific IgG levels in sera and MLN after challenge, if compared to the control vaccine virus [39].

The limitation of the study is that we did not assess functional activity of the vaccine-induced M2e antibodies It is supposed that the M2e antibody can mediate protection via the antibody-dependent cellular cytotoxicity (ADCC) or complement-dependent cytotoxicity mechanisms [40,41,42], however direct measurement of ADCC activity in ferrets is challenging due to the lack of ferret-specific reagents [43]. Another limitation is that we did not assess the levels of mucosal IgA responses after vaccination. It is known that the nasal IgA antibody can play a role in reducing the load of challenge virus in the URT, since IgA antibody are naturally cross-neutralizing [44]. However, in the current study, the decrease in viral titers in the URT did not correlate with the virus clearance from the lungs, suggesting that the sIgA antibody were not the main immune factors involved in the enhanced cross-protection. Nevertheless, the mucosal IgA and T-cell immune responses induced after LAIV immunization should also contribute to the overall protective potential of the vaccine.

It is interesting to compare the protective effect of two different universal LAIV strategies. We previously developed a panel of reassortant LAIV strains expressing stem-based chimeric HA molecules and evaluated them in sequential immunization regimens in ferrets [18]. Both ferret studies used the same S.A. H1N1 challenge virus, which made it possible to compare the protective effect of the induced immune responses. Strikingly, the levels of stalk-HA-reactive antibodies induced by a three-dose sequential immunization with cHA-based LAIVs were not sufficient to significantly reduced viral titers and lung pathology after the H1N1 challenge, and the inclusion of a NP gene from the H1N1 virus into LAIV genome was required to achieve statistically significant reduction in viral pulmonary titers and clinical signs of disease [18]. In contrast, the two-dose immunization with the LAIV+4M2e candidates induced strong M2e-specific antibody responses with cross-protective potential. One possible explanation for these discrepant results is that the cHA-based LAIVs replicated poorly in the ferrets’ upper respiratory tract, whereas the M2e-based LAIV candidates were highly infective and animals shed the vaccine viruses up to day 4 post inoculation, and such enhanced replicative activity could promote robust M2e-specific antibody responses. Another possibility is that the stalk-HA-targeted and the M2e antibodies differ by their avidity and Fc-mediated functional activities, however a side-by-side experiment is required to confirm this assumption.

Besides the induction of balanced adaptive immune responses, the M2e-based universal vaccine based on a licensed LAIV backbone has more advantages over other M2e-based approaches, such as an adjuvant-free technology and an intranasal route of administration, whereas the majority of other M2e-based vaccine prototypes are injectable and require adjuvants to boost potent M2e-specific antibody responses [5]. Recent advances in influenza reverse genetics technology allow rapid generation of recombinant LAIV reassortant viruses with updated major antigenic determinants, including the design of the chimeric HA+4M2e gene to match currently circulating seasonal influenza viruses. However, if vaccine-induced broad immunity is to be sustained, regular antigen update will not be required. Although the current study did not assess the duration of the induced immune responses, a recent cHA-based study of LAIV candidates revealed the persistence of HA stem-reactive antibodies in immunized ferrets for at least one year [45].

## 5. Conclusions

Given the safety profile and improved cross-protective potential, the LAIV/HA+4M2e vaccine warrants its further evaluation in a phase I clinical trial.

## Data Availability

The data presented in this study are available on request from the corresponding author.

## Supplementary Materials

The following are available online at https://www.mdpi.com/article/10.3390/v13071280/s1, Figure S1: Gating strategy for the assessment of antigen-specific T-cell immune responses in ferret splenocytes, Figure S2: Histopathological evaluation of respiratory tissues of ferrets immunized with study vaccines or placebo, Figure S3: Serum IgG antibody immune responses after vaccination, Figure S4: Histopathological analysis of ferret respiratory tissues after challenge, Figure S5: T-cell immune responses in immunized ferrets challenged with heterologous influenza virus, Figure S6: Correlation analysis of viral loads in respiratory tissues of immunized ferrets post-challenge with different immunological outcomes.
