# Supplementary material for: Universal Live-Attenuated Influenza Vaccine Candidates Expressing Multiple M2e Epitopes Protect Ferrets against a High-Dose Heterologous Virus Challenge
Source: Viruses. 2021 Jun 30;13(7):1280. doi: 10.3390/v13071280 (PMC8310119; doi:10.3390/v13071280)
Supplement: Supplementary file 1 [file viruses-13-01280-s001.zip › viruses-1281957-SI.pdf]

viruses

Supplementary Materials for paper "Universal live-attenuated influenza vaccine candidates expressing multiple M2e epitopes protect ferrets against a high-dose heterologous virus challenge"

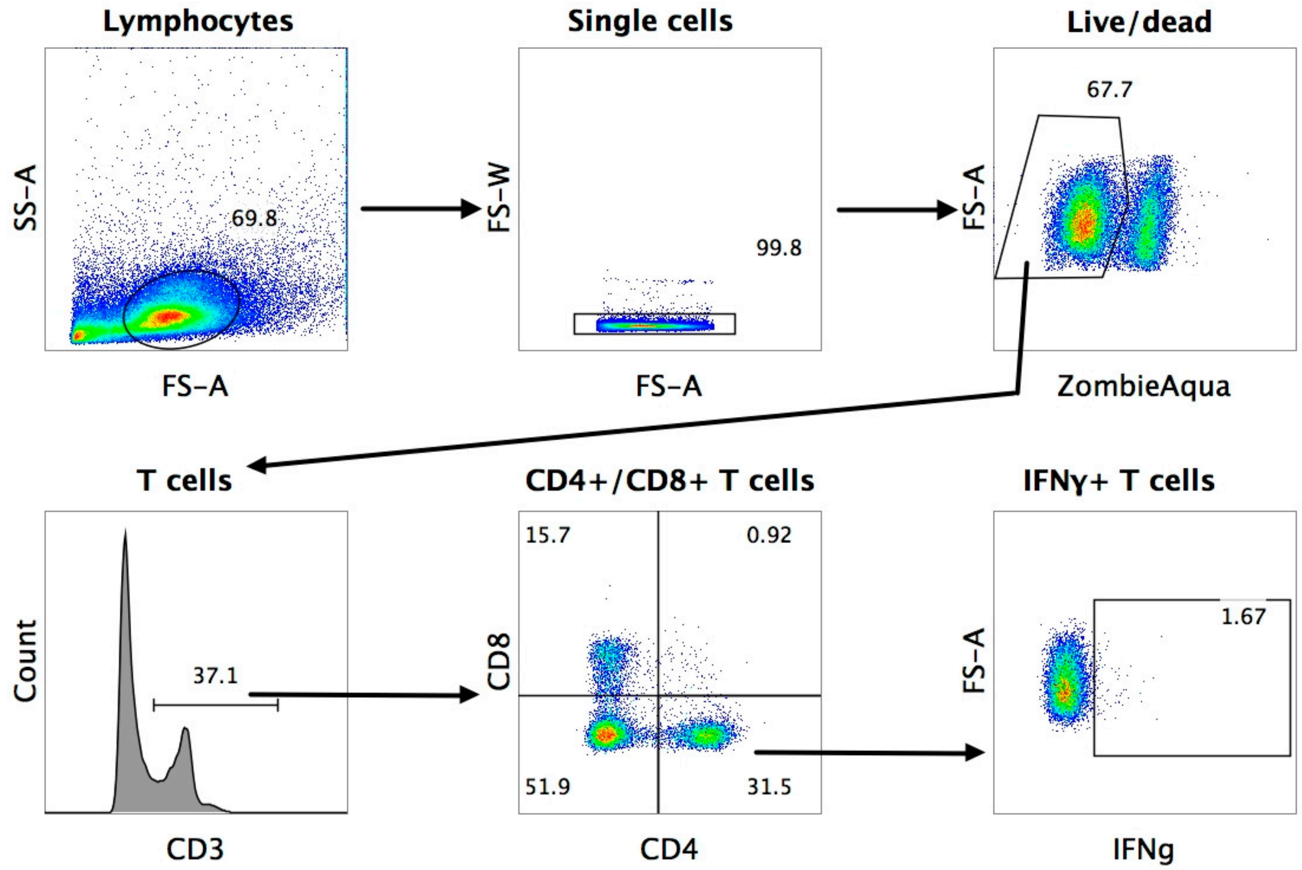

Figure S1. Gating strategy for the assessment of antigen-specific T-cell immune responses in ferret splenocytes.

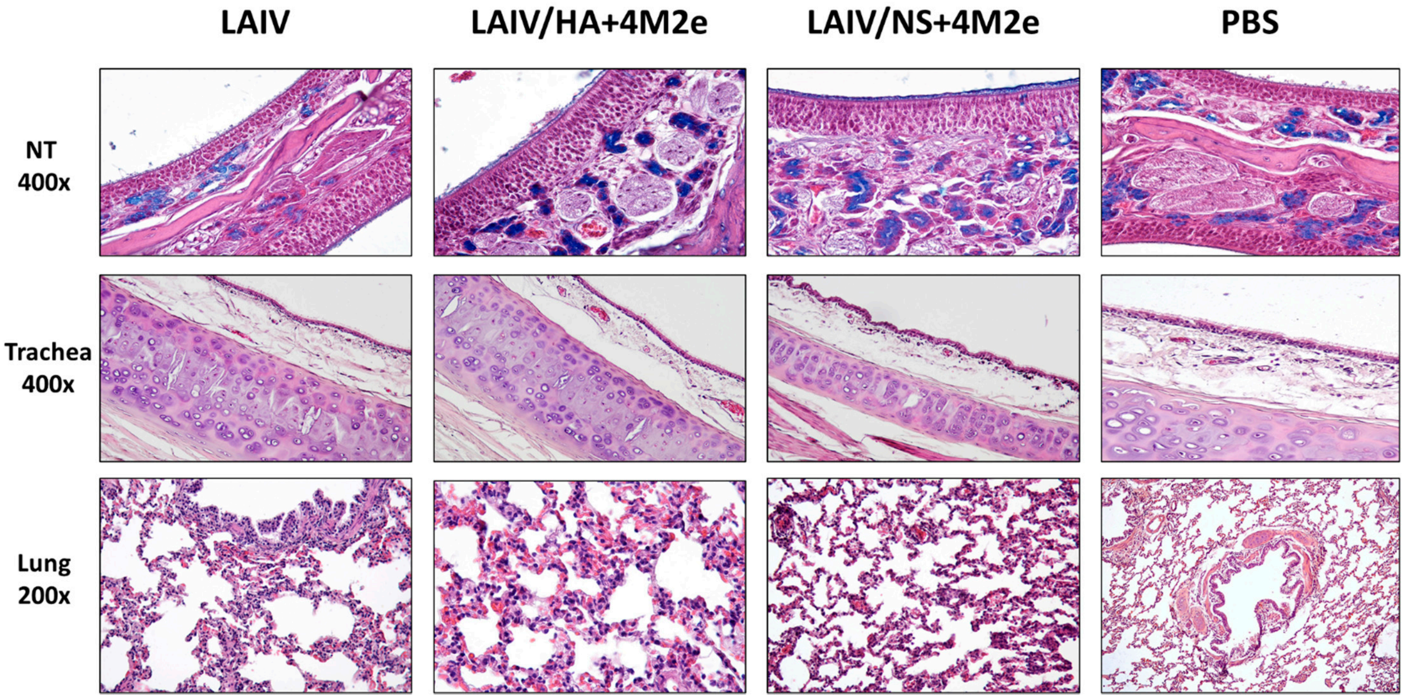

Figure S2. Histopathological evaluation of respiratory tissues of ferrets immunized with study vaccines or placebo. Nasal turbinates, trachea and lung tissues were collected from three animals on day 7 after the second vaccine dose. Tissues were fixed in a 10% buffered formalin solution and subjected to standard histoprocessing. Prepared sections were stained with hematoxylin and eosin, as well as with alcian blue dyes (for nasal turbinates). Histological preparations were examined in a light microscope under indicated magnification.

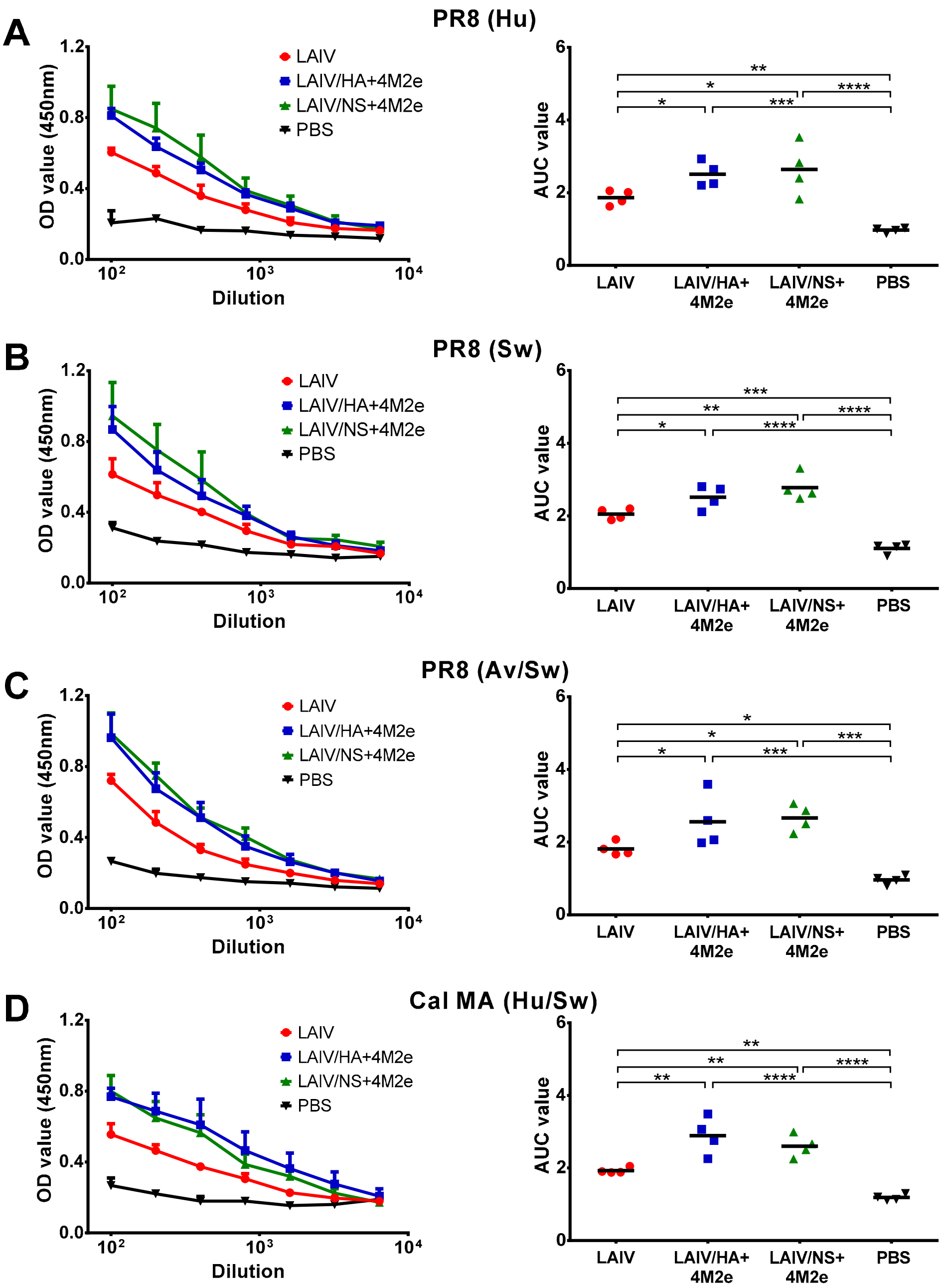

Figure S3. Serum IgG antibody immune responses after vaccination. Groups of four ferrets were immunized intranasally with study vaccine or PBS, twice with a 3-week interval. Serum samples were collected 21 days post-boost and antibody binding was assessed in cell ELISA using MDCK cells infected with indicated virus as an antigen. **A.** A/PR8 virus carrying M gene of A/Aichi/2/1968 (H3N2) (M2 of human lineage). **B.** A/PR8 virus carrying engineered M gene of swine lineage. **C.** A/PR8 virus with M gene of A/duck/Potsdam/1402-6/1986 (H5N2) virus (M2e of avian/swine lineage). **D.** Cal MA H1N1 (M2e of human/swine lineage). Left panel shows mean±SD of OD_{450} values for each serum dilution. Right panel shows the area under the OD450 curve for each individual animal. Data were analyzed by one-way ANOVA with Tukey's post-hoc multiple analyses test. *p<0.05; **p<0.01; ***p<0.001; ****p<0.0001.

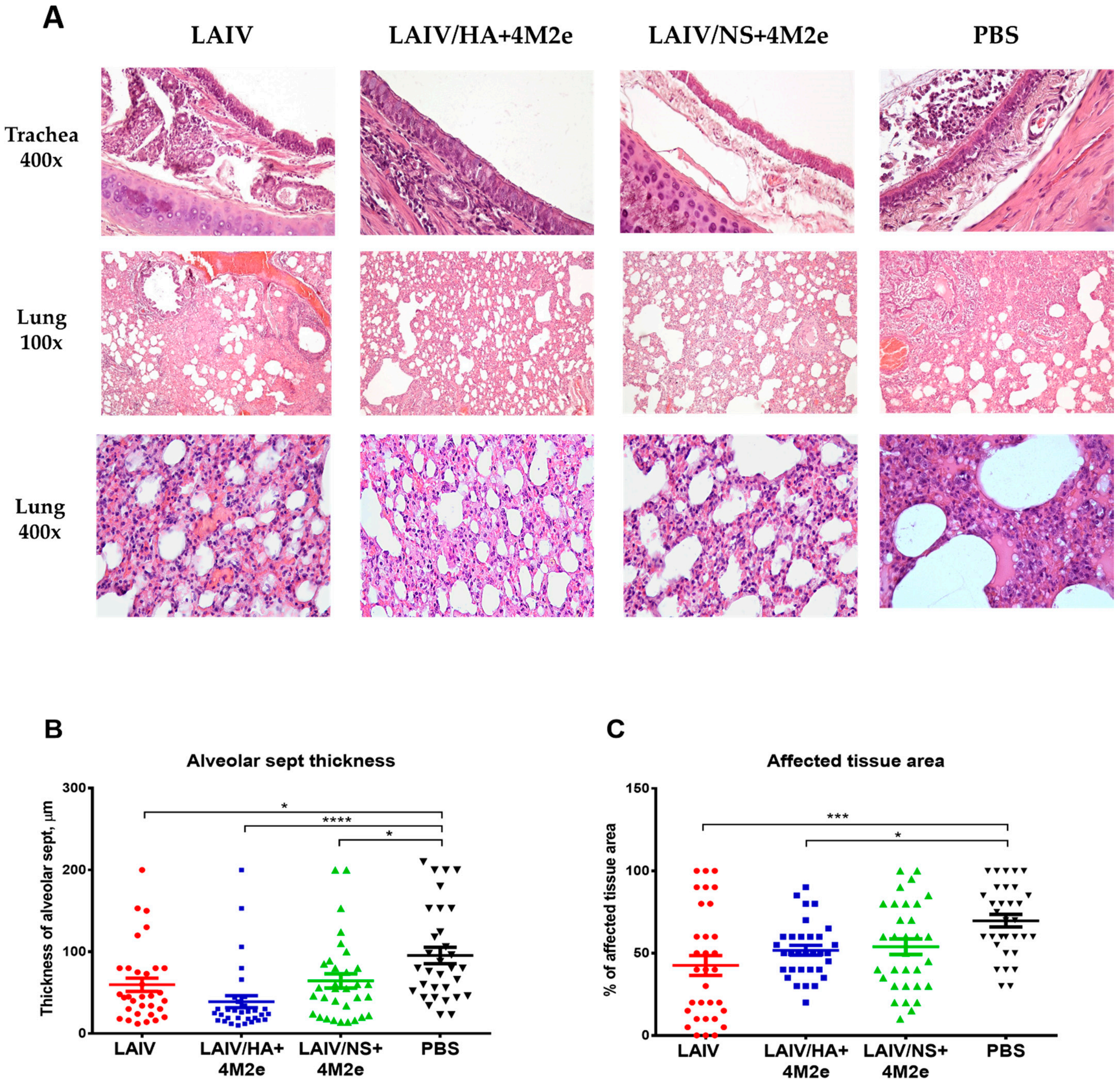

Figure S4. Histopathological analysis of ferret respiratory tissues after challenge. **A.** Trachea and lung tissues were collected from four animals on day 5 after challenge. Tissues were fixed in a 10% buffered formalin solution and subjected to standard histoprocessing. Prepared sections were stained with hematoxylin and eosin and examined in a light microscope under indicated magnification. **B.** Alveolar septum thickening assessed by a microscope with calibrated objectives. **C.** The area of affected lung tissue in % of all field area, as measures by a microscope at 200x magnification with a calibrated objectives micrometer taking into account 15-40% compression of wet tissues due to fixation and sample histoprocessing. Data were analyzed by one-way ANOVA with Tukey's post-hoc multiple analyses test (right panel). *p<0.05; ***p<0.001, ****p<0.0001.

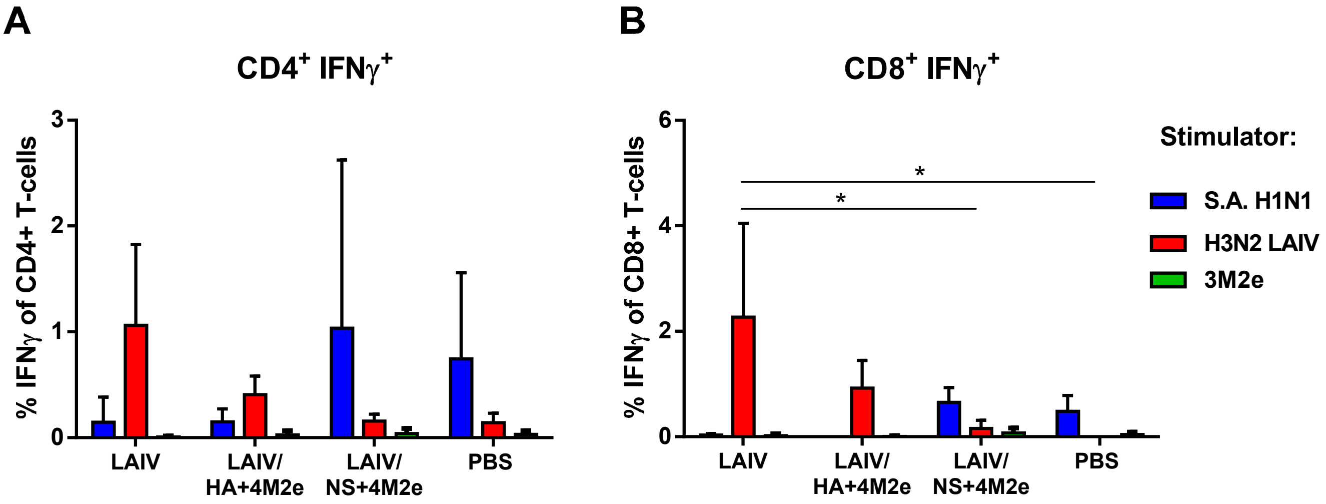

Figure S5. T-cell immune responses in immunized ferrets challenged with heterologous influenza virus. Spleen tissues were collected from four animals on day 5 after challenge and isolated splenocytes were stimulated in vitro with H3N2 or H1N1 whole viruses, or with 3M2e protein. Antigen-specific CD4 (**A**) and CD8 (**B**) responses were assessed by intracellular staining of IFNγ and analyzed by flow cytometry. Data were analyzed by two-way ANOVA with Tukey's post-hoc multiple analyses test. *$p<0.05$.

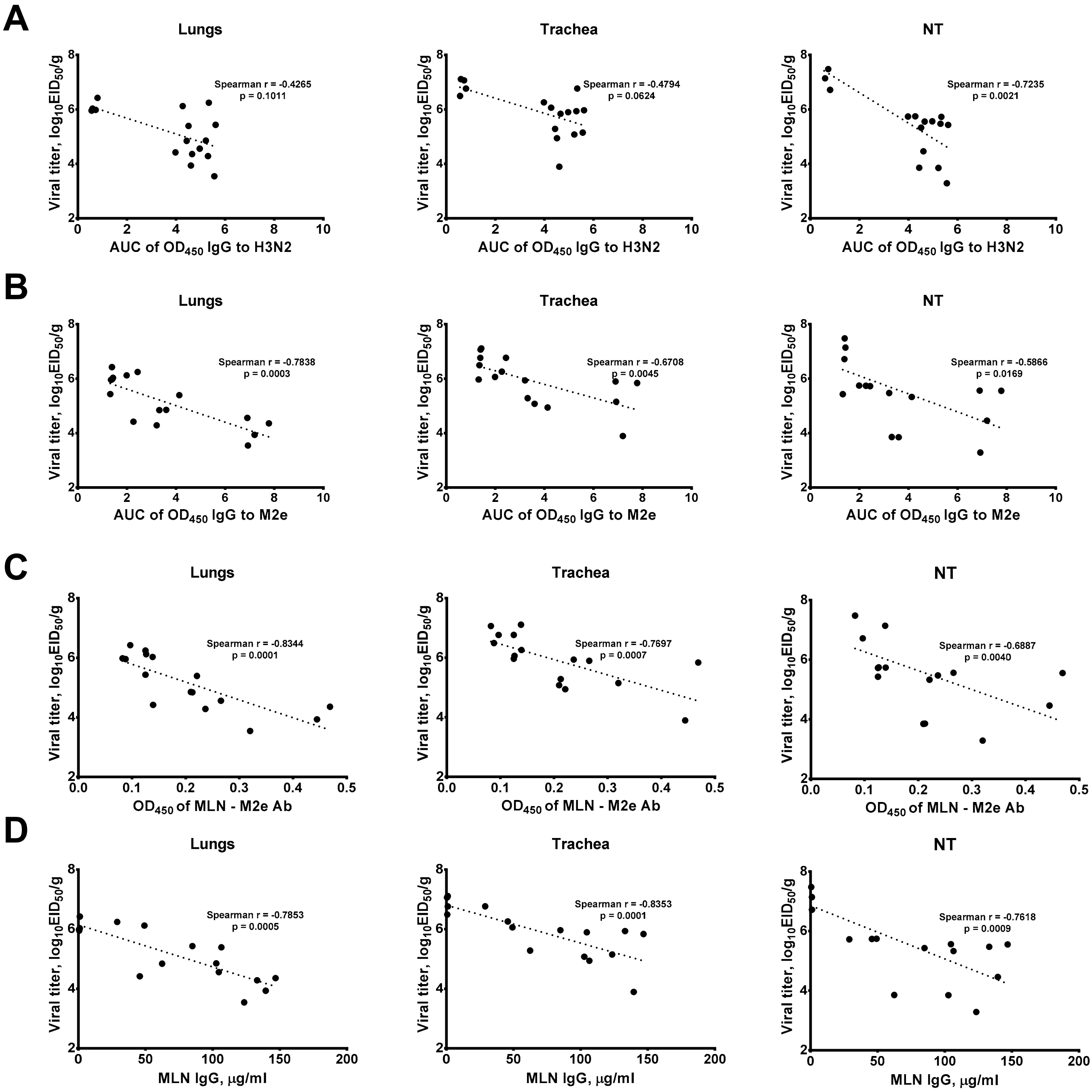

Figure S6. Correlation analysis of viral loads in respiratory tissues of immunized ferrets post-challenge with different immunological outcomes: **A.** H3N2 whole virus-specific serum IgG levels three weeks after the second immunization. **B.** M2e-specific IgG levels three weeks after the second immunization. **C.** M2e-specific IgG antibody produced by MLN cells collected on day 5 post-challenge. **D.** Concentration of total IgG antibody produced by MLN cells collected on day 5 post-challenge after their 6-day culturing in vitro.
